# Motor and Dexterity Assessments as Predictors of Functional Independence in Post-stroke Patients

**DOI:** 10.7759/cureus.104558

**Published:** 2026-03-02

**Authors:** Selma Šabanagić-Hajrić, Nevena Mahmutbegovic, Emir Hasanbegovic, Adnan Al-Tawil, Amina Al-Tawil, Denijal Mukinovic

**Affiliations:** 1 Neurology Clinic, University Clinical Center Sarajevo, Sarajevo, BIH; 2 Department of Radiology, Private Clinic Dr Al-Tawil, Sarajevo, BIH; 3 Department of Internal Medicine/Cardiology, Private Clinic Dr Al-Tawil, Sarajevo, BIH; 4 Internal Medicine Clinic, Department of Nephrology, Dialysis and Kidney Transplantation, University Clinical Center Tuzla, Tuzla, BIH

**Keywords:** activities of daily living, barthel index, berg balance scale, functional independence, modified rankin scale

## Abstract

Introduction: Stroke is a leading cause of long-term disability, and early functional prognostication is essential for individualized rehabilitation planning.

Objective: The objective of the study is to examine the association between bedside motor and dexterity tests and standard outcome measures, and to evaluate their predictive value for functional independence in post-stroke patients.

Materials and methods: This observational study was conducted at the Neurology Clinic, University Clinical Center Sarajevo, Sarajevo, Bosnia and Herzegovina, and included 61 patients with either ischemic or hemorrhagic stroke. Sociodemographic and clinical data were collected. Bedside functional assessments comprised the National Institutes of Health Stroke Scale (NIHSS), the Motor Assessment Scale (MAS), the Nine-Hole Peg Test (9-HPT) for upper limb dexterity, and the Berg Balance Scale (BBS). Functional outcomes were evaluated using the Barthel Index and the modified Rankin Scale (mRS) at admission and discharge. Associations between bedside assessments and functional outcomes were analyzed using correlation analyses, and independent predictors of functional independence were identified using multivariable linear regression models.

Results: The mean age of participants was 74.8 ± 5.5 years, and 73.8% had ischemic stroke. Functional independence improved during hospitalization, with the Barthel Index increasing from 65.3 ± 22.9 at admission to 72.1 ± 26.5 at discharge. In multivariable regression analysis, the MAS emerged as the strongest independent predictor of functional independence measured by the Barthel Index (R² = 0.88, β = 1.49; p < 0.001). Performance on the 9-HPT using the non-dominant hand provided additional independent predictive value (β = -0.28; p = 0.048), while dominant-hand dexterity and balance performance were not significant predictors in the adjusted model.

Conclusions: Simple bedside motor and dexterity assessments provide clinically relevant prognostic information in the early post-stroke phase. Their integration into routine clinical practice may enhance functional prognostication and support individualized rehabilitation strategies.

## Introduction

Stroke remains one of the leading causes of chronic neurological disability worldwide, with notable individual, societal, and economic consequences [1]. Survivors frequently experience persistent impairment in motor control, balance, coordination, and upper limb function that compromises independence in activities of daily living (ADLs) [2]. These functional limitations often remain despite novel advances in acute stroke care and represent a major target for long-term rehabilitation [3]. Early and accurate assessment of functional ability is therefore critical, not only to quantify the extent of post-stroke impairment but also to predict the course of recovery and guide individualized rehabilitation strategies [4].

A variety of standardized tools have been developed for bedside evaluation of stroke-related functional impairment. Among them, the Nine-Hole Peg Test (9-HPT) provides a rapid and objective measure of upper limb dexterity [5], the Motor Assessment Scale (MAS) evaluates a broad range of motor activities [6], and the Berg Balance Scale (BBS) quantifies balance and postural control [7]. These performance-based measures are widely used in both research and clinical practice due to their feasibility and sensitivity to functional change [8]. However, their predictive value with respect to global outcome measures such as the Barthel Index and the modified Rankin Scale (mRS)-which remain the gold standards for assessing functional independence and disability in stroke populations-requires further evaluation [9,10]. Therefore, the objective of this study was to examine the association between bedside functional tests (9-HPT, MAS, and BBS) and standard outcome measures (Barthel Index and mRS), and to explore their potential predictive role in determining functional independence among post-stroke patients.

## Materials and methods

This observational study was conducted at the Neurology Clinic, University Clinical Center Sarajevo, Sarajevo, Bosnia and Herzegovina, and included 61 patients with either ischemic or hemorrhagic stroke. The study protocol was reviewed and approved by the institutional Ethics Committee (Ethics Committee approval No. 06-04-9-33476.6), and written informed consent was obtained from all participants.

Sample size and sampling

This study used a consecutive convenience sample of all eligible patients admitted to the Neurology Clinic, University Clinical Center Sarajevo, during the study period. Due to the exploratory observational design, no formal a priori sample size calculation was performed.

Inclusion criteria were age ≥ 18, confirmed diagnosis of stroke, stable clinical status, and ability to perform functional tests. Exclusion criteria comprised severe aphasia/cognitive impairment, presence of acute complications, or comorbidities significantly affecting motor function. Patients with missing key functional data were excluded from the final analysis.

Sociodemographic data (age, sex), clinical characteristics (stroke type, National Institutes of Health Stroke Scale (NIHSS) score at admission and discharge), and functional assessments were collected. Patients were assessed during the subacute in-hospital phase, once they achieved medical stability and were considered ready for discharge planning. The primary outcome measures, therefore, reflect short-term functional independence at hospital discharge. As the primary focus of the study was functional status at discharge, duration of hospitalization was not included as an analytical variable.

Functional evaluation included the 9-HPT (dominant and non-dominant hand) for upper limb dexterity, the MAS, the BBS, the Barthel Index, and the mRS. All clinical and functional assessments were performed by board-certified neurologists trained in standardized stroke outcome evaluation.

All assessment tools were used as clinical outcome measures; no copyrighted questionnaires were reproduced in full. Each scale was cited according to its original validation publication. Due to the observational in-hospital design, blinding of outcome assessors was not feasible.

Statistical analysis

Statistical analyses were performed using standard procedures for observational clinical research. Descriptive statistics were applied to summarize baseline demographic and clinical characteristics. Categorical variables were presented as numbers and percentages, whereas continuous variables were expressed as mean ± standard deviation (SD), median values with interquartile range (IQR), and minimum-maximum range, depending on the distribution of the data.

The associations between bedside functional assessments (9-HPT, MAS, and BBS) and outcome measures (Barthel Index and mRS) were examined using correlation analyses. Pearson’s correlation coefficient was used for normally distributed variables, while Spearman’s rho was applied when distributional assumptions were not met. Prior to regression modeling, all functional variables were inspected for multicollinearity and linearity assumptions.

To identify independent predictors of functional outcome, multivariable linear regression models were constructed with the Barthel Index at discharge and mRS as dependent variables. Variables demonstrating significant associations in univariate analyses were entered into the regression models using the enter method. Although mRS is an ordinal scale, linear regression was applied for consistency and interpretability of effect sizes, as commonly used in clinical stroke outcome research. The coefficient of determination (R²) was used to quantify the proportion of explained variance. The number of predictors included in the regression models was limited in relation to the sample size to reduce the risk of overfitting.

For analytical consistency, 9-HPT values exceeding the upper time limit of the test were recoded as 101 s, representing maximal impairment and permitting inclusion in the models. Statistical significance was set at p < 0.05 for all analyses. Multicollinearity was assessed using variance inflation factors (VIFs), with all values below the acceptable threshold (<5), indicating no significant multicollinearity among predictors. All statistical procedures were conducted using IBM SPSS Statistics, version 26 (IBM Corp., Armonk, NY, USA, 2019).

## Results

Baseline characteristics

A total of 61 post-stroke patients were included in the analysis, with a mean age of 74.8 ± 5.5 years; 55.7% were male. The majority experienced ischemic stroke (73.8%), whereas 26.2% had hemorrhagic stroke. The mean NIHSS score at admission was 9.2 ± 4.8, decreasing to 7.7 ± 6.2 at discharge, indicating overall neurological improvement during hospitalization (Table 1).

**Table 1:** Baseline sociodemographic and clinical characteristics of the study population (N = 61) Data are presented as n (%) for categorical variables and mean ± SD for continuous variables. NIHSS: National Institutes of Health Stroke Scale; SD: standard deviation

Characteristic	N (%)/mean ± SD
Sex (male/female)	34 (55.7%)/27 (44.3%)
Age (years), mean ± SD	74.8 ± 5.5
Stroke type (ischemic/hemorrhagic)	45 (73.8%)/16 (26.2%)
NIHSS at admission, mean ± SD	9.2 ± 4.8
NIHSS at discharge, mean ± SD	7.7 ± 6.2

Functional independence

Baseline functional independence assessed by the Barthel Index (T0) demonstrated substantial impairment with a mean score of 65.3 ± 22.9. By discharge (T1), scores improved to 72.1 ± 26.5, reflecting variable but clinically meaningful recovery. Performance on the 9-HPT indicated prolonged upper limb dexterity times for both the dominant (65.7 ± 26.3 s) and non-dominant (70.7 ± 25.7 s) hands. Motor performance measured using the MAS yielded a mean of 34.4 ± 14.0 points, while balance ability assessed by the BBS averaged 33.6 ± 17.8 points. Global disability, as measured by the mRS, averaged 2.5 ± 1.3 points (Table 2).

**Table 2:** Descriptive statistics of functional outcome measures Values are presented as mean ± SD, median with interquartile range (IQR), and minimum-maximum. Maximum possible scores and ranges: Barthel Index (0-100), MAS (0-56), Berg Balance Scale (0-56), and mRS (0-6); 9-HPT is expressed as time in seconds (lower values indicate better performance). 9-HPT: Nine-Hole Peg Test; MAS: Motor Assessment Scale; SD: standard deviation

Variable	Mean ± SD	Median (IQR)	Min-Max
Barthel Index at admission (T0)	65.3 ± 22.9	75.0 (50.0-85.0)	15-95
Barthel Index at discharge (T1)	72.1 ± 26.5	80.0 (60.0-95.0)	20-100
9-HPT dominant hand (s)	65.7 ± 26.3	66.9 (38.9-88.1)	25.36-101
9-HPT non-dominant hand (s)	70.7 ± 25.7	74.2 (43.7-99.2)	31.99-101
MAS total score	34.4 ± 14.0	40.0 (30.0-48.0)	0-56
Berg Balance Scale	33.6 ± 17.8	40.0 (18.0-46.0)	0-56
Modified Rankin Scale (mRS)	2.5 ± 1.3	2 (2-3)	0-6

Multivariable regression analysis demonstrated high explanatory power (R² = 0.88), with MAS emerging as the strongest independent predictor of functional independence (β = +1.49; p < 0.001). Additionally, non-dominant hand 9-HPT performance contributed independently to the model, albeit with a smaller effect (β = -0.28; p = 0.048). Neither dominant hand 9-HPT nor BBS retained significance in the adjusted model (Table 3).

**Table 3:** Multivariable regression models for functional outcome In adjusted models, MAS was the strongest independent predictor of both functional independence and global disability, with the non-dominant hand 9-HPT contributing additional predictive value only for the Barthel Index. Multivariable linear regression analysis (enter method). Standardized β coefficients are presented. MAS: Motor Assessment Scale; 9-HPT: Nine-Hole Peg Test

Dependent variable	R²	Significant predictors
Barthel Index (T1)	0.88	MAS (β = +1.49; p < 0.001), 9-HPT non-dominant (β = –0.28; p = 0.048)
Modified Rankin Scale (mRS)	0.82	MAS (β = –0.063; p < 0.001)

## Discussion

The present study evaluated the predictive role of functional tests-including the 9-HPT, MAS, and BBS-for independence and disability outcomes among patients in the early post-stroke phase. Our findings demonstrated that standardized bedside measures provide strong prognostic information regarding recovery potential and functional outcomes. In contrast to assumptions that isolated fine motor performance might dominate prognostic models, the present analysis revealed that MAS emerged as the strongest independent predictor of both functional independence and global disability, as measured by the Barthel Index and by the mRS. The prognostic relevance of MAS is supported by results of two prospective multicenter studies [11,12]. Brauer et al. reported that higher MAS scores significantly increased the likelihood of being discharged home rather than to institutional care, and Tucak et al. found that initial MAS scores were strongly associated with duration of hospitalization and functional mobility [11,12].

Performance on the 9-HPT of the non-dominant hand showed additional predictive value for the Barthel Index, whereas neither the dominant hand 9-HPT nor BBS retained independent significance after multivariable adjustment. Recent literature consistently confirms that functional independence after stroke is underpinned by broader motor abilities, which is in line with our results [13]. Studies assessing the psychometric properties of these tools indicate high reliability and clinical applicability in stroke populations [13]. For example, the BBS has shown excellent test-retest reliability in hemiparetic patients (intraclass correlation coefficient (ICC) ≈ 0.98), while MAS and 9-HPT have also demonstrated high reliability (ICC ≥ 0.95 for MAS, ICC ≈ 0.85 for 9-HPT in post-stroke hand paresis) [14]. Such reliability supports their clinical use in prognostic evaluation, as results can be considered both reproducible and clinically valid.

Although BBS demonstrated strong associations with functional outcomes in univariate analyses, its effect was no longer significant once MAS was included in the regression model. Our findings are opposite to other research; Louie and Eng reported that BBS was the only independent predictor of unassisted walking recovery, with each additional point increasing the odds of regaining ambulation (OR ≈ 1.11) [15]. Threshold values have also been established; for instance, BBS ≥ 29 at admission predicts community ambulation speed (≥0.8 m/s) with high accuracy (area under the curve (AUC) = 0.88) [15]. While previous findings point to the relevance of balance for functional mobility, our results suggest that its prognostic value diminishes when global motor capacity is accounted for.

In our study, MAS emerged as the strongest independent predictor in our regression models. A recent Danish study reported that each additional point on the MAS score at admission increased the odds of being discharged home rather than to institutional care (OR = 1.14, 95% CI 1.04-1.25). Patients scoring ≥24 at admission were 17 times more likely to return home, with high sensitivity (91.7%) and moderate specificity (68%) [16]. These findings confirm the predictive validity of MAS for discharge destination.

Upper limb dexterity, as quantified by 9-HPT, also contributed to the prognostic profile, particularly through the non-dominant hand, which independently predicted Barthel Index scores. This result reintegrates fine motor assessments in identifying residual upper-limb deficits that affect ADLs, even when global motor scales already demonstrate strong effects. However, evidence in the literature is mixed. Lin et al. found that 9-HPT performance one month after stroke did not reliably predict functional independence at six months, suggesting limited long-term prognostic utility [17]. It is possible that 9-HPT reflects a specific motor domain-fine manual dexterity-without fully capturing compensatory strategies or overall independence. Nevertheless, in our short-term outcome assessment, 9-HPT remained clinically useful as a supplementary predictor, indicating its value in early rehabilitation planning, particularly when combined with other functional measures.

Despite the strengths of these functional instruments, several studies have reported inconsistent predictive validity [18,19]. First, the timing of test administration strongly affects results. In the acute phase, many patients cannot perform BBS due to severe deficits, while in later rehabilitation phases, high scores may hide remaining impairments [18,19]. Second, functional independence is multidimensional, relying not only on motor skills but also on cognition, perception, and psychosocial factors [18,19]. Dong et al. demonstrated that combining motor scales with cognitive screening substantially improved outcome prediction in mild stroke [20]. Finally, clinical observations indicate that good scores on mobility tests may reflect compensatory strategies rather than genuine neurological recovery, limiting their predictive specificity [21].

This study has several limitations. The relatively small sample size and single-center design limit the generalizability of results. The observational study design with short-term in-hospital follow-up precludes causal inference and does not allow evaluation of long-term predictive validity. The relatively narrow age distribution and exclusion of patients with severe aphasia may limit generalizability to older and more severely impaired stroke populations. In addition, ceiling and floor effects may have influenced the discriminatory power of certain tests. Future multicenter studies with larger samples and longitudinal follow-up are warranted to confirm these results, establish clinically relevant cut-off values, and explore whether combining motor, balance, and cognitive assessments enhances prognostic accuracy.

## Conclusions

In conclusion, simple bedside motor and dexterity assessments provide clinically relevant prognostic information in the early post-stroke phase. The MAS emerged as the strongest independent predictor of both functional independence and global disability, underscoring the central role of global motor function in early recovery. Upper limb dexterity, particularly non-dominant hand performance on the 9-HPT, contributed additional predictive value for ADL. These findings suggest that structured bedside motor assessments may support early identification of patients at risk for reduced functional independence; however, external validation in larger, multicenter studies is warranted before routine clinical implementation.
